# From the Paper to the Tablet: On the Design of an AR-Based Tool for the Inspection of Pre-Fab Buildings. Preliminary Results of the SIRAE Project

**DOI:** 10.3390/s18041262

**Published:** 2018-04-19

**Authors:** Cristina Portalés, Sergio Casas, Jesús Gimeno, Marcos Fernández, Montse Poza

**Affiliations:** 1 Institute of Robotics and Information and Communication Technologies (IRTIC), Universitat de València, 46980 València, Spain; sergio.casas@uv.es (S.C.); jesus.gimeno@uv.es (J.G.); marcos.fernandez@uv.es (M.F.); 2 DRAGADOS, S.A., 28050 Madrid, Spain; mpozav@dragados.com

**Keywords:** Augmented Reality, pre-fab buildings, inspection, energy efficiency

## Abstract

Energy-efficient Buildings (EeB) are demanded in today’s constructions, fulfilling the requirements for green cities. Pre-fab buildings, which are modularly fully-built in factories, are a good example of this. Although this kind of building is quite new, the in situ inspection is documented using traditional tools, mainly based on paper annotations. Thus, the inspection process is not taking advantage of new technologies. In this paper, we present the preliminary results of the SIRAE project that aims to provide an Augmented Reality (AR) tool that can seamlessly aid in the regular processes of pre-fab building inspections to detect and eliminate the possible existing quality and energy efficiency deviations. In this regards, we show a description of the current inspection process and how an interactive tool can be designed and adapted to it. Our first results show the design and implementation of our tool, which is highly interactive and involves AR visualizations and 3D data-gathering, allowing the inspectors to quickly manage it without altering the way the inspection process is done. First trials on a real environment show that the tool is promising for massive inspection processes.

## 1. Introduction

It is well known that there is a gap between the predicted and the measured energy performance of buildings, once they are constructed [1]. This can be critical for energy-efficient buildings (EeB), as they are demanded to be of high-energy performance. Defects in building construction and/or materials can be a source of energy loss. This can be reduced by inspecting the building and their components at different stages of the construction process, thus correcting any possible deviation from the design phase. Inspection is a required and standard process in the assessment of building quality, including energy efficiency. For instance, in [2] insight is given into the use of a standard for building condition assessment. As described therein, the decision mode of the inspector to determine the stage of a building can be as follows: the inspector, after dealing with the inspection following the standard processes according to the regulations, decides about the defects, their importance (minor, serious, or critical), their intensity (low, middle, or high) and their extent (incidentally, locally, regularly, frequently, or generally). The extent and the intensity of a defect combined with the importance of the defect leads to a condition rating (critical, serious, and minor defects). Based on this, the inspector decides about the building stage.

Traditionally, the inspection process has been documented with paper support, and the signature of the inspector was the only proof regarding the building condition stage. However, new technologies, such as Augmented Reality (AR), can aid in the inspection process, for instance, by allowing a more reliable documentation of the inspection (e.g., by acquiring imaging proofs of any possible defects or the absence of them) or by guiding the inspector through his/her work.

AR is a computer-based technology by which virtual data can be added to the user’s perception of the real world. It simultaneously combines real and virtual objects which are registered in a 3D space, and it is interactive in real-time [3]. Most AR applications deal with visual stimuli by merging virtual objects with images of the real world, which are computationally generated in real-time. The image of the real world usually comes from a camera stream and it is visualized on a screen merged with the virtual information. Nevertheless, other displays can also be used to show only the virtual information, while users see the real world through the display (see-through displays) or projected on them (AR mirrors [4,5]). On the other hand, as a result of the rapid development of smart mobile devices, AR systems based on smartphones or tablets represent the most common setup. This makes the AR paradigm almost ubiquitous, and it is perhaps one of the main reasons why AR has become so popular in recent years.

Mobile AR provides many potential benefits since it can be used in a wide variety of scenarios: medical treatment and rehabilitation [6], learning and training [7,8], engineering and construction [9,10], entertainment [11], cultural heritage [12,13], etc. However, this popularity does not cause all AR applications to be successfully used in real environments, especially in construction environments, where conditions are particularly harsh (electromagnetic noise, dust, changing light conditions, etc.) and robust solutions are difficult to build. Factories, construction sites and other hostile environments represent a challenge for the use of AR systems. One successful example of the use of AR in a complex hostile environment is presented in [14], where AR and VR are used to help improve supervision and maintenance tasks in the Large Hadron Collider (LHC) at CERN, Geneva. An interesting feature of this work is the use of a gamma camera to capture emitted radiation and show this vital information for workers on top of visible images. Within the inspection process, these smart monitoring visual techniques are important elements and mobile devices could play a vital role, as in [15].

In recent years some research exists that deals with novel interactive tools to aid the inspector in the process of the assessment of building quality and its documentation, some of them including AR technology. For instance, the work presented in [16] showed the potential use of AR in architectural construction, inspection, and renovation, by means of X-ray AR vision. In [17] an AR system is presented for urban environments, where the positioning of the user in real-time is achieved with the use of a GPS. Interesting from this paper is that the system allows performing annotations. In [18], an AR application was presented for industrial building environments that enabled comparing as-planned documentation with the factory that was actually built. The application ran on a mobile device, where the augmented views showed overlaying 3D models on top of a video image of the real environment. More recently, in [19] a method based on an energy performance AR environment was presented, dealing with the automated analysis and visualization of deviations between buildings’ actual and simulated energy performances. In [20] the issues and needs of current defect management practices in the construction industry were investigated, also presenting a conceptual system framework for construction defect management that integrated ontology and AR with building information modelling. Another example is reported in [21] where a mobile defect management AR application was presented, which enabled workers and managers to automatically detect dimension errors and omissions on the jobsite.

Other works exist that use AR to bridge the gap between the digital and the real world in the construction sector [22], also aiding in the buildings’ facility control and maintenance [23,24]. In [25] a mobile AR application is presented for BIM registration, where positioning in real-time is achieved by means of a Kinect sensor. Finally, in [26] an interesting work is presented for on-site acoustic inspection, where the measurement data is post-processed and converted into a three-dimensional numerical model of the acoustic indicators, which is integrated afterwards into an AR application.

However, the specific case of the inspection of pre-fab EeB with interactive tools—for inspecting both the individual components and the entire constructive modules—has not been explored yet by the research community, to the best of our knowledge. Additionally, not all the proposed systems allow making annotations on the virtual assets or retrieving in situ information of a different nature about the constructive elements (e.g., images, shape acquisition by means of clouds of three-dimensional points, etc.).

In this paper, we present the preliminary results of the SIRAE project that aims at detecting and, thus, eliminating the quality and energy efficiency deviations between the designed and the constructed buildings, focusing on pre-fab buildings. To that end, we are developing a new computational interactive tool for automatizing the process and documentation of the inspection in pre-fab buildings (individual components and building modules), while giving new functionalities to the inspectors based on digital documentation (images and 3D clouds of points) and AR visualizations. The tool embeds an entire inspection process of pre-fab buildings and, thus, its further use in the massive inspection of these buildings is promising. The major challenges we deal with in the project are: (i) to build a computational tool that integrates AR to assist the inspector while keeping unaltered the procedures of the inspection process in order to avoid the need of new training and to ensure consistency with the existing inspection regulations; (ii) to make a tool usable in a (noisy and hostile) construction environment, which, in the case of pre-fab buildings, also includes a factory, that involves user positioning in real-time inside buildings; (iii) to make all the processes highly interactive while collecting data of different natures (3D clouds of points, images, annotations, drawings, etc.) that must be georeferenced and related to each other.

The project started in September 2015 and will last until December 2018. At this stage, we have the first version of our prototype fully operationally tested by an inspector in situ and by other inspectors in a laboratory environment. We are currently working on the final version, which will be validated in a real environment next summer. In this paper, we communicate the results of the first version.

The rest of the paper is structured as follows. In Section 2, pre-fab buildings are introduced, giving an insight of their inspection process. In Section 3, the interactive tool is explained in detail, showing the involved hardware technology and software solution. In Section 4, the preliminary results are discussed and further work is outlined. Finally, the conclusion section summarizes our main findings.

## 2. The Modular Building System

The modular building system consists on the construction of buildings, of one or more stories, based on three-dimensional modules of metal structures and welded joints. These modules can be vertically and/or horizontally assembled, thus resulting in an entire building. The modules are prefabricated in a factory and incorporate all the enclosures, partitions, finishes, and necessary installations. Once fabricated, they are transported to the corresponding location and assembled, according to the established order, and supported on a foundation previously executed in situ. On site, the modules are connected to the foundation and to each other, giving continuity to the enclosures, partitions and finishes between modules, completing the installations, and making the necessary connections between modules, finally giving rise to a single building.

The main features of this modular system are its versatility to achieve any type of finish, adapting to each project, and the control of the execution thanks to the industrialization of the construction process. Being a transportable construction and constructed by the addition of modules, the buildings can be expanded, transformed, or moved to another site, with a flexibility that goes beyond the possibilities of the traditional construction process.

**Figure 1:**
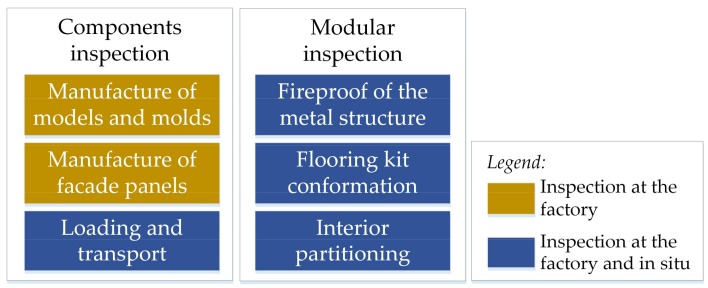
Inspection of components and modules of pre-fab buildings.

Regarding to the inspection process, the modules are both inspected in the factory and in situ at different stages of their construction, both the manufactured components and the complete modules. Some of the items belonging to an inspection process are outlined in Figure 1. For all of the inspected items, the inspector fulfils a questionnaire on a paper support, annotating measurements, observations and any kind of possible defect found and its characteristics. If the inspected item passes the inspection test, the inspector gives his/her conformity by signing the document. Otherwise, the item is replaced, discarded, or corrected, depending on the kind of defect, and the inspection process on the new item is performed again. It has to be added that, depending on the project, the allowed deviations in the measurements might differ, but the specific requirements for each modular construction do always follow the current regulations, including those related to energy saving [27,28,29].

## 3. Description of the System

The inspection followed in the SIRAE project follows the same inspection procedure as indicated above, but gives the inspector new interactive computational tools to document the process easier and better in real-time. In this sense, the computational tool embeds all the paper-based questionnaires and additionally allows the acquisition of images and 3D data, as well as providing with AR views. Among others, the AR technology provides [30] the ability to enhance reality, while allowing a seamless interaction between real and virtual objects. Based on this, the inspection process can be improved without altering the existing procedures.

In the following subsections, the hardware technology and the computational tool used in SIRAE are explained in detail.

### 3.1. Hardware Technology

**Figure 2:**
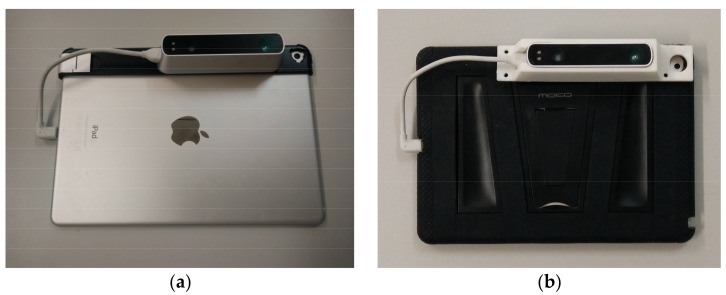
Hardware used in the SIRAE project consisting of an iPad and a Structure Sensor (**a**); and the devices protected with cases (**b**).

The hardware set-up used for the developed application consists of an iPad equipped with an Occiptal’s Structure Sensor [31] (Figure 2a). This hardware is suitable to support the needs of the project, which requires a hardware capable of supporting the following aspects: visualization of the questionnaires related to the inspection process, possibility to make annotations, possibility to build AR visualizations, information capture (images and 3D cloud of points), robustness, and enough autonomy to cover a full working day.

In this sense, the iPad has an autonomy of 10 h, suitable to cover a full working day. Its dimensions are sufficient to correctly visualize the data shown to the operator (such as the questionnaires), while not excessive, so it offers mobility and does not constitute a burden for the operator. The screen is tactile—so handwriting is available—and it has enough luminosity to work outdoors. It also integrates an RGB camera, which can be used to gather data (images), to build the AR scenes, and to aid in the real-time positioning. In this last case, other integrated sensors in the iPad (e.g., accelerometers) can be used. Additionally, its own construction makes this a fairly sealed device, suitable for its use in factories or construction sites.

Regarding to the AR visualizations requirement, three fundamental aspects are needed: to compute the point of view in real-time, to show the virtual content blended and aligned with the real environment, and to be interactive. With the iPad technology, virtual and real objects can be blended, and the device also allows interaction in real time. To capture the point of view there are numerous solutions based on image processing (such as ARCore, ARKit or Vuforia). Therefore, only with the use of an iPad can the point of view can be computed. However, this type of solution needs to capture textured images in which to recognize enough characteristics for the calculations of the point of view. In our specific case, the prefabricated modules are usually of homogeneous colors, so this type of solutions does not work correctly, so an additional technology is needed—we use the Structure Sensor to deal with this.

The Structure Sensor is a scanner that acquires depth information in the form of range images from which 3D clouds of points with RGB and IR values are computed, after the scanner is calibrated with the iPad’s integrated camera. Range images are obtained after projecting a pattern of infrared light that is observed by its infrared camera, a technology similar to the Kinect. With this device, it is, therefore, not necessary to have a highly-textured environment, and it works correctly in prefabricated module environments. Additionally, this technology is able to gather homogeneous and dense 3D clouds of points, a fact that is difficult to achieve with the aforementioned technologies.

On the other hand, the Structure Sensor also fulfils other requirements, as it is lightweight and has measuring ranges and accuracies aligned with the requirements of a building inspection. The scanner has an autonomy of 3 to 4 h in full scanning, according to its technical specifications. Therefore, it is more than enough for the inspection process, as the scanner will only be used at specific moments, mainly if the inspector decides to document the shape of an object or if the AR window is active. The working distances of the scanner range from 40 cm to 3.5 m, which are suited to inspect modular building systems. The accuracy of the sensor depends on the observed depth. For a distance of 1.5 m, the depth precision is better than 1 cm.

In order to be used during a real inspection process, the devices were protected with respective cases (Figure 2b). The case for the iPad is a standard one, made with rubber and with a screensaver, so it is protected against possible blows or falls. On the other hand, for the depth sensor we designed a specific case that protected its contour, and then we materialized it with a 3D printer.

### 3.2. Computational Tool

**Figure 3:**
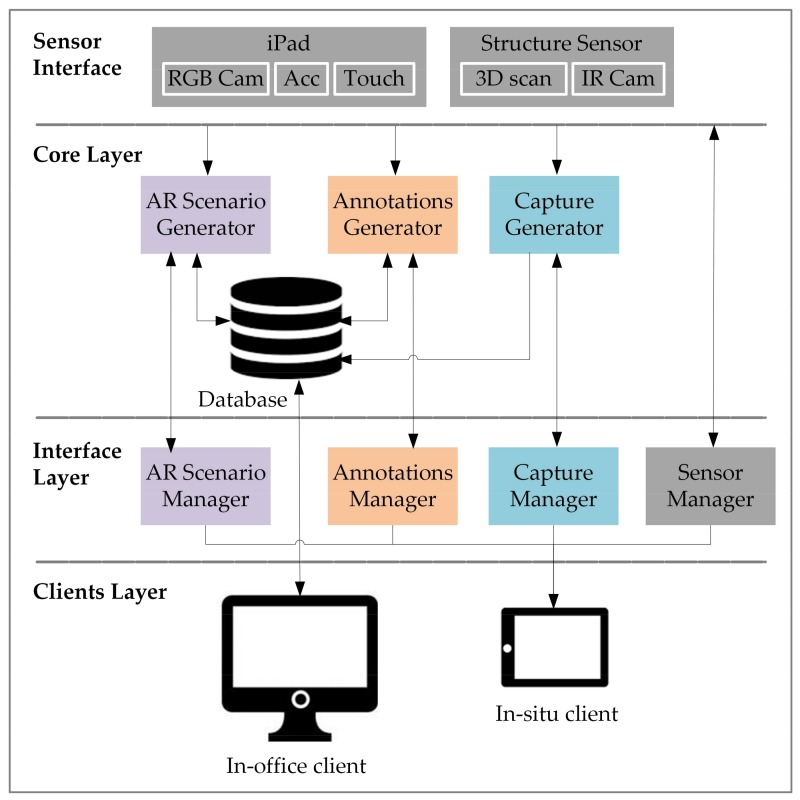
Software architecture in SIRAE, where the sensor interface and the three software layers are depicted.

The interactive tool has been implemented in Unity 3D [32]. It consists of a set of windows that embed all the inspection items shown in Figure 1 (components inspection and modular inspection). In Figure 3, the architecture of the software is schematized. Conceptually, the software consists of three layers: the core layer, the interface layer, and the clients’ layer. The core layer embeds the internal procedures for the generation of the AR scenario, the annotations, and the capture of data (images and 3D clouds of points). The procedures retrieve data from the hardware, having access to the different sensors embedded in them. These procedures are also connected to a database, in such a way that the captured information and the annotations are stored there, but also information from the database can be retrieved in real-time. For instance, to build the AR scenario, virtual models of the original planned structures (built by the in-office client) can be loaded in real-time, so any deviations from the original design can be easily depicted. The interface layer consists of a set of windows for the AR scenario visualization, the annotations that inspectors can do in real-time (text, numbers, handwriting, labelling, etc.), the capturing of data and the managing of sensors’ settings (iPad/Structue Sensor calibration, screen brightness, etc.). These four kind of windows are available through the iPad’s tactile screen (the in situ client), which belongs to the clients layer.

To begin the inspection, the inspector introduces manually the project identifier, so the characteristics of the project are loaded into the application. Then, the inspector chooses between one of the different items to inspect (e.g., manufacture of GRC panels) and then adds a new registry and manually enters the lot identifier.

**Figure 4:**
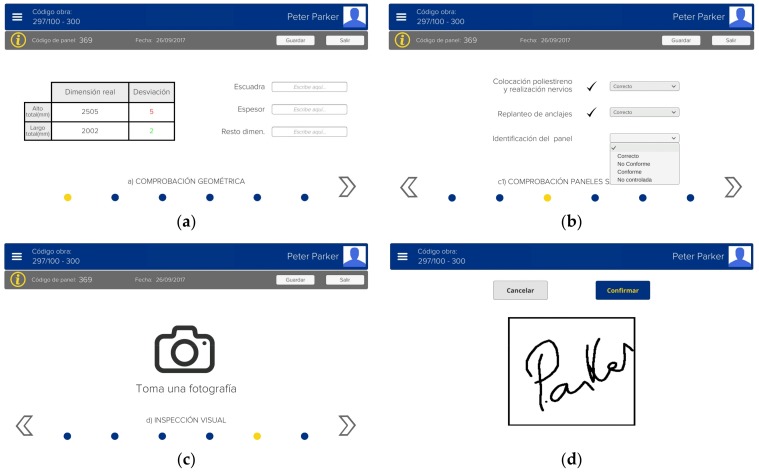
Examples of windows of the interface layer, for the item “manufacture of GRC panels”: (**a**) geometric checking, where the inspector can insert numeric values; (**b**) panels checking, where the inspector can choose one option from drop-down menus; (**c**) visual inspection, where an image can be taken; and (**d**) signature, where the inspector can give the conformity to the inspection process by hand-signing it.

An example is given in Figure 4, where different windows of the application (interface layer) are shown for the item “manufacture of façade panels” (recall Figure 1). The application shows the inspector all the steps required to fulfil the inspection, providing him/her with all the functions and choices that the traditional inspection process has, including the possibility to take notes. However, differently from the traditional inspection, in our system annotations are digitally performed and can be related to digital content, such as a plan of the building as designed, images taken during the inspection, or gathered 3D clouds of points. In this way, the inspector can add measures taken with another device (e.g., a measuring tape or a theodolite), check the inspected points, add comments, etc. As the project specifications are loaded beforehand, if one of the measures taken by the inspector is out of tolerance, it appears reflected in the tool in red color, thus advising the inspector that a defect is present (Figure 4a). Once the item is fully inspected, and if no defects are found, the inspector agrees by giving his/her signature (Figure 4d).

**Figure 5:**
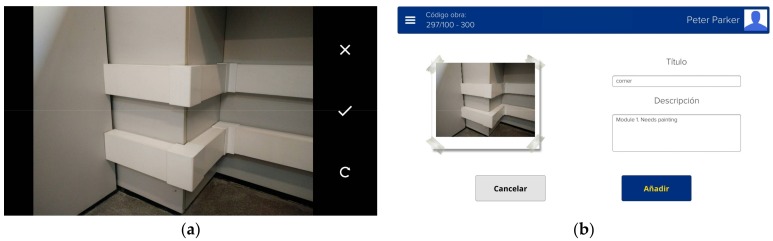
Image acquisition: (**a**) a captured image, with accept/cancel/rotate buttons; and (**b**) a captured image with related information, written by the inspector during the process; the add button adds the image and the comments to the database.

At different parts of the inspection process, the functionality of acquiring data, both images and 3D clouds of points with texture information (RGB and IR values), is integrated. An example of a button for acquiring images can be seen in Figure 4c in the form of a camera icon, where in Figure 5a the acquisition and storage of an image is seen, while Figure 5b shows an example of text fields related to the acquired image that the inspectors can use to document it. The acquired data is stored in the database with an identifier that relates them unequivocally with a specific project and a specific item/object inside a specific lot. Thus, this information can be recovered at any time and can be used to generate summaries of the inspection process.

On the other hand, the AR visualization can be activated when inspecting a whole module rather than individual components. It allows having a view of the designed module superimposed to the real scenario. As it has a closed view (virtual models consist of complete rooms where users are inside), the visualization is done by adding an alpha value to the virtual model. Users can choose the alpha value, so they can interactively change between only depicting the real scenario, only depicting the virtual model, or merging both views in real-time. To load the corresponding virtual model, an AR marker is placed in the module at a specific location. When the inspector points to the AR marker, the virtual model appears, 3D-registered and aligned to the real one, based on the camera positioning with respect to the AR marker. In order to track this marker, we have integrated the Vuforia library in the Unity 3D project. Once the virtual model is loaded, navigation is achieved by means of the Structure Sensor. The Structure Sensor SDK is provided for iOS and it can be integrated in Unity 3D. As explained before, it uses a pattern of infrared light projected on the real world to capture the shape of surfaces. Based on these shapes, the sensor is able to compute the external orientation of the device (displacements and rotations) relative to the previous position, which we transfer to the virtual camera in the Unity 3D project, taking into account the initial position of the AR maker, so the virtual and real worlds are aligned while navigating.

## 4. Discussion and Future Work

**Figure 6:**
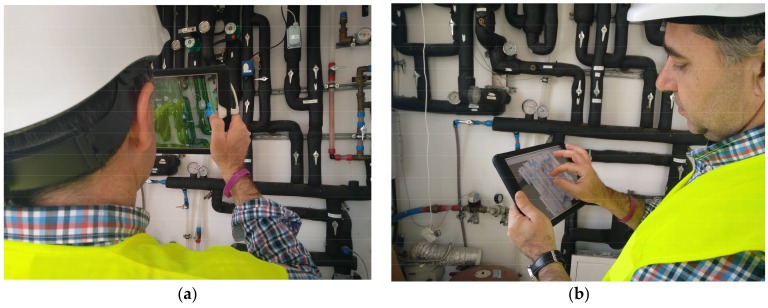
An inspector making use of the interactive tool in the process of acquiring 3D data of a defective piece: (**a**) acquiring the shape of the piece; and (**b**) inspecting the acquired piece.

The interactive tool has been designed in close collaboration with inspectors with expertise in performing the inspection of modular buildings in order to ensure its further usability by them. In this section, we show some images of an inspection process with the interactive tool in a real scenario (Figure 6), also commenting on the lessons learned and future work. The trials took place in a pre-fab buildings’ factory in Seville in autumn 2017. At the factory, one inspector tested the application in situ. After that, he and two other inspectors tested the application at the office, with the aim to give us feedback on the usability of the application and the accuracy of the integration of the inspection steps within our solution.

The inspector testing the application at the factory found the tool very intuitive. He could easily follow all the questionnaires without the need to ask us on how to use the tool. He only needed a few explanations about the new capabilities related to the 3D setups, both to record data and to show a 3D shape of the designed module in an AR window. Regarding all the new inspection capabilities, the inspector found really valuable that the tool could take images, and that those images were directly stored in a relational database, so they could be related to a specific location inside the module (i.e., they are geo-referenced). Additionally, he pointed out the great new capabilities that further documenting the possible defects (or the absence of them) with 3D data and texture information (both RGB and IR images) provides, as some projects include volumetric shapes that are difficult to fully record in a single image.

After the inspection with the developed tool, and brainstorming with the other two inspectors, a set of issues were identified that could become useful to further improve the interactive tool. In the first place, they commented about the possibility of directly loading the project and lot data without having to type the identifier. In this sense, we are currently implementing a QR-code identifier inside the application, so this step will be fully automated. Secondly, they commented about the possibility of interactively measuring distances once 3D data is acquired. This functionality is already integrated in the Structure Sensor’s SDK, so we will make this available inside our application. Additionally, we will allow the inspectors to choose between seeing the measurements on top of the acquired 3D model, or directly on top of the real view of the camera while moving around the object, as it is possible to keep the positioning of the user relative to the object in real-time thanks to the integrated SLAM techniques in the depth sensor SDK. Finally, they commented about having digital access to the plans of the module in order to make annotations on it. We are also implementing this functionality, so each time a project is loaded, the plans will be available in image or PDF formats, allowing handwriting on top of them. The annotated plans will also be stored in the relational database, so they can be recovered at any time and can be used to generate summaries of the inspection process as well.

It is also worth mentioning the benefits that this new digital inspection tool can bring in terms of time saving. Though images and 3D data acquisition can take some time, we believe that the overall time needed to perform the inspection process with the new tool should be similar or less than that of a traditional documentation process. Indeed, in paper-based documentation, when a defect is found, the inspector has to give his/her non-conformity, describing the kind and nature of the defect. This text information can be quickly replaced by an image or a quick scan of the defect. Additionally, as in the interactive tool, all the gathered information is digitally stored in a relational database, the different kinds of data are connected (and geo-referenced), greatly reducing the post-work at the office and the danger of losing information because of damaged paper, wrong manual transmission to a digital form, or wrong information matching. This, and other aspects, will be further demonstrated once the development of the tool is completed.

## 5. Conclusions

Inspection is a required process for assessing a building’s quality. Though there is typically a gap in energy saving between designed and constructed buildings, correcting them from any possible deviation during the construction phase reduces the gap. A common way of documenting the inspection process is using conventional tools, mainly based on paper support. In this paper, we have presented the first outcomes of the SIRAE project, aimed to provide an interactive tool that can seamlessly aid in the processes of pre-fab building inspections, allowing the inspectors to digitally document the process with images and 3D data, besides the traditional measurements and annotations. The designed tool is based on the AR technology and allows the acquisition of images and 3D clouds of points, while annotations can be also done. Additionally, the application takes into consideration the traditional inspection process, so inspectors do not need to learn new ways of doing their work, focusing on the process itself rather than on the provided new technology. The first trials carried out in a real environment show the capabilities of our tool to be included in regular inspection processes. We also believe that the methodology that we have developed can be extrapolated to the inspection of other types of buildings, although the interactive tool should be adapted if the inspection steps vary. In this sense, we are currently working in a generic tool from which customized GUIs can be derived, what would make this possible.
